# Heterologous Overexpression of Apple *MdKING1* Promotes Fruit Ripening in Tomato

**DOI:** 10.3390/plants12152848

**Published:** 2023-08-02

**Authors:** Qianyu Yue, Xinyue Yang, Pengda Cheng, Jieqiang He, Wenyun Shen, Yixuan Li, Fengwang Ma, Chundong Niu, Qingmei Guan

**Affiliations:** 1 Shenzhen Research Institute, Northwest A&F University, Shenzhen 518000, China; yue_qianyu@163.com; 2 State Key Laboratory of Crop Stress Biology for Arid Areas/Shaanxi Key Laboratory of Apple, College of Horticulture, Northwest A&F University, Yangling, Xianyang 712100, China; yang13963533651@126.com (X.Y.); cheng17360736713@163.com (P.C.); jieqiang.he.dawn@gmail.com (J.H.); swy2019060109@nwafu.edu.cn (W.S.); liyixuan20@nwafu.edu.cn (Y.L.); fwm64@nwsuaf.edu.cn (F.M.)

**Keywords:** *MdKING1*, tomato, fruit ripening, ethylene, carotenoid

## Abstract

Fruit ripening is governed by a complex regulatory network, and ethylene plays an important role in this process. MdKING1 is a γ subunit of SNF1-related protein kinases (SnRKs), but the function was unclear. Here, we characterized the role of *MdKING1* during fruit ripening, which can promote fruit ripening through the ethylene pathway. Our findings reveal that *MdKING1* has higher expression in early-ripening cultivars than late-ripening during the early stage of apple fruit development, and its transcription level significantly increased during apple fruit ripening. Overexpression of *MdKING1* (*MdKING1* OE) in tomatoes could promote early ripening of fruits, with the increase in ethylene content and the loss of fruit firmness. Ethylene inhibitor treatment could delay the fruit ripening of both *MdKING1* OE and WT fruits. However, *MdKING1* OE fruits turned fruit ripe faster, with an increase in carotenoid content compared with WT. In addition, the expression of genes involved in ethylene biosynthesis (*SlACO1*, *SlACS2*, and *SlACS4*), carotenoid biosynthesis (*SlPSY1* and *SlGgpps2a*), and fruit firmness regulation (*SlPG2a*, *SlPL*, and *SlCEL2*) was also increased in the fruits of *MdKING1* OE plants. In conclusion, our results suggest that *MdKING1* plays a key role in promoting tomato fruit ripening, thus providing a theoretical basis for apple fruit quality improvement by genetic engineering in the future.

## 1. Introduction

Ripening, the final stage of fruit development, is governed by an elaborate regulatory network and is of practical importance to the human diet [1]. Fruit quality develops gradually during ripening and is precisely regulated. However, fruit ripening is a complex process that involves a series of biochemical and physiological changes, including decreased firmness of the fruits, increased ethylene content, and accumulation of carotenoids, which affect the development of fruit qualities such as color, aroma, flavor, and texture [2]. Moreover, fruit ripening can be affected by a variety of factors, like genetics, environmental conditions, and hormone levels [3,4], among which the regulation of plant hormones plays an important role, such as ethylene, auxins, abscisic acid, jasmonates, and brassinosteroids [5].

According to ethylene emissions and respiration rates during fruit ripening, fruits can be divided into climacteric fruits (apple, tomato, banana, mango) and non-climacteric fruit (strawberry, grape, cherry, pineapple) [6,7]. From previous studies, abscisic acid (ABA) mainly regulates non-climacteric fruit ripening [8], while ethylene plays a key role in the ripening regulation of climacteric fruits [9]. Ethylene, as the most important phytohormone affecting fruit ripening, is regulated by biosynthesis and signal-transduction pathways in fruit [5,10]. Ethylene biosynthesis reaction consists of two steps, S-adenosyl-l-methionine (SAM) is transformed into 1-aminocyclopropane-1-carboxylic (ACC) by ACC synthase (ACS), and then ACC is catalyzed into ethylene formation by ACC oxidase (ACO) [11]. In apple fruit, *MdACS6*, *MdACS3a*, and *MdACS1* are the most important *ACS* genes that play an important role in fruit ripening and ethylene biosynthesis [12,13,14]. Additionally, inhibition of *ACS2* and *ACS4* gene expression in tomato fruit delayed fruit ripening by reducing ethylene production [15,16]. Chen’s [17] studies show that ACO acts as an accelerator in fruit ripening [17]. In the ethylene signal-transduction pathway, ethylene first binds to the receptor, and then the signal can be transduced into the downstream genes [18]. Ethylene receptors include three families that have been identified, including ethylene resistant (ETR), ethylene response sensor (ERS), and ethylene insensitive 4 (EIN4) [5].

SNF1-related protein kinase 1 (SnRK1) in plants is an ortholog of yeast sucrose non-fermenting 1 (SNF1) and mammalian AMP-activated protein kinase (AMPK), and they function as heterotrimeric complexes, including an α-catalytic subunit (KINα) and two regulatory subunits (KINβ and KINγ) [19,20]. In plants, a βγ subunit (KINβγ), which seems to be a hybrid β and γ subunit, confers canonical γ subunit functionality, but the γ subunit (KINγ) is not directly involved in SnRK1 signaling [21]. In in vitro analysis, KINγ has the potential to regulate the activity of SNF1-related protein kinase 2s (SnRK2s) [22], but the biological function of KINγ is still unclear. In Arabidopsis, overexpression of SnRK1 could increase the sensitivity of Arabidopsis to glucose and ABA, and the addition of glucose during ABA treatment further enhanced the sensitivity of SnRK1 overexpression plants to ABA [23]. Three SnRK2 subfamily genes, SnRK2.2/SnRK2.3/SnRK2.6, could regulate cuticle development through the ABA pathway [24]. Regardless of SnRK1 or SnRK2, they all play an important role in plant development. The ABA signaling pathways interacted with SnRK signaling pathways to regulate plant growth and metabolism. The phosphorylation level of MdSnRK2.4/2.9 (two MdSnRK2-I members) significantly increased throughout apple fruit development and ripening, MdSnRK2-I increased *MdACO1* expression, and MdACS1 protein stability promoted ethylene production in apple fruits [25]. LcSnRK1α modulated litchi fruit senescence by interacting with and phosphorylating two transcription factors, LcbZIP1 and LcbZIP3 [26]. In tomatoes, overexpression of *MhSnRK1* and *PpSnRK1α* promotes fruit ripening [27,28,29]. SnRK1 phosphorylated transcription factor bZIP39 to regulate sorbitol metabolism in “Royal Gala” apple [30]. However, it is not clear whether the KINγ can affect fruit ripening.

Apple has the characteristics of self-incompatibility, high genome heterozygosity, and long childhood. Therefore, the functional verification of genes related to fruit traits in apples still lags behind other crops [31]. Tomato has emerged as a well-defined model system for dissecting the ripening mechanism of climacteric fruit [10], accompanied by a short developmental cycle, while apple is also a typical climacteric fruit. Therefore, we can use tomatoes as background material to verify the regulatory functions of candidate genes related to fruit development in apples through heterologous transformation. Here, we found that *MdKING1*, which belongs to a SnRK1 γ subunit (KINγ), could play an important role in fruit ripening. Then, we cloned and overexpressed *MdKING1* in tomato plants. *MdKING1* OE plants exhibited a significantly higher ethylene and carotenoid content and accelerated fruit ripening. Meanwhile, ethylene synthesis, carotenoid synthesis, and fruit firmness-related gene expression are significantly upregulated in *MdKING1* OE plants. In conclusion, this study reveals the positive regulatory function of *MdKING1* in fruit ripening and provides a theoretical basis for fruit quality improvement and new cultivar breeding.

## 2. Results

### 2.1. Identification and Expression Analysis of MdKING1

**Figure 1:**
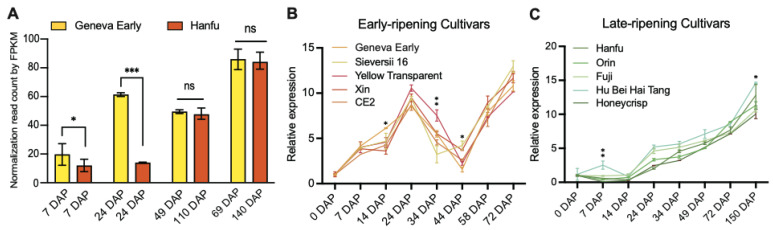
The expression patterns of *MD06G1142700* are consistent with fruit development in apples. (**A**) Expression levels of *MdKING1* during fruit development at different periods in apple cultivars “Geneva Early” and “Hanfu”. (**B**) *MD06G1142700* expression levels in early-ripening cultivars. (**C**) *MD06G1142700* expression levels in late-ripening cultivars. Fragments per kilobase per million mapped fragments (FPKM) of *MD06G1142700* in different fruit development stages and different tissues were retrieved from the previous data. Error bars indicate SD (*n* = 3). Statistical significance analysis was determined using Student’s two-tailed *t* test (* *p* ≤ 0.05, ** *p* ≤ 0.01, *** *p* ≤ 0.001; ns, no significance).

**Figure 2:**
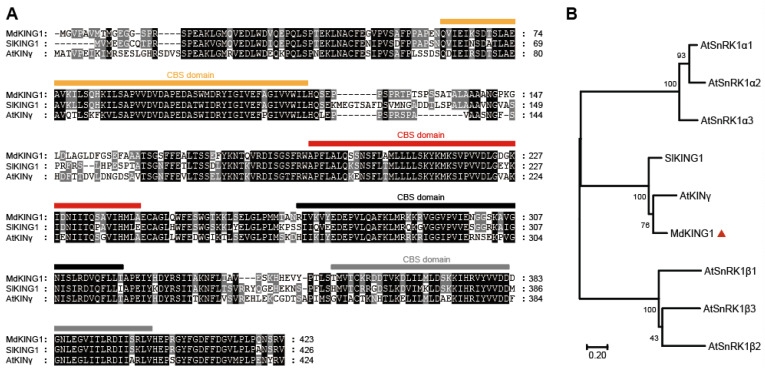
MdKING1 (MD06G1142700) is the homologous of SnRK1 γ subunit in apple. (**A**) Sequence alignment of KING1 (KINγ) proteins in apple, tomato, and Arabidopsis; the predicted CBS domains are indicated by squares. (**B**) Phylogenetic analysis of KING1 (KINγ) proteins, SlKING protein, and other SnRK1 subunits in Arabidopsis. The numbers at the nodes of the branches represent bootstrap values. Md, *Malus domestica*; Sl, *Solanum lycopersicum* L.; At, *Arabidopsis thaliana*. Accession numbers are as follows: MdKING1 (MD06G1142700), SlKING1 (Solyc03g111310.3.1), AtSnRKα1 (At3g01090), AtSnRKα2 (At3g29160), AtSnRKα3 (At5g394400), AtSnRKβ1 (At5g21170), AtSnRKβ2 (At4g16360), AtSnRKβ3 (At2g28060), and AtKINγ (AT3G48530). The orange, red, black, and gray bars represent the 4 CBS domains. The red triangle remarks the protein of MdKING1.

We previously identified that the homolog of *MD06G1142700* was associated with the development of apple (*Malus* c *domestica* cv.) fruits [32], in which the expression of early-cultivar “Geneva Early” was significantly higher than that of late-cultivar “Hanfu” at 7 and 24 days after pollination (DAP). During the process of apple fruit development to maturity, the expression of *MD06G1142700* maintained an increasing trend and reached the peak at the picking stage (Figure 1A). We also selected five varieties in the early-ripening and late-ripening accessions for verification, and the results showed that the *MD06G1142700* gene in the early-ripening accessions was still expressed at a higher level than that in the late-ripening at 24 DAP (Figure 1B,C). Sequence analysis showed this gene contains a putative coding region of 1272 bp, which encodes 432 amino acids with a predicted molecular weight 47.06 kDa. BLASTP analysis of protein sequence alignment showed that it had 69% sequence identity with KINγ in Arabidopsis and 74.29% with tomatoes, which contained four tandem cystathionine β–synthase (CBS) motifs (Figure 2A). Furthermore, the phylogenetic analysis showed that *MD06G1142700* is homologous to AtKINγ (Figure 2B); we therefore named it MdKING1. These data suggested that the function of *MdKING1* may be related to fruit ripening in apples.

### 2.2. Overexpression of MdKING1 Accelerates Fruit Ripening in Tomato

**Figure 3:**
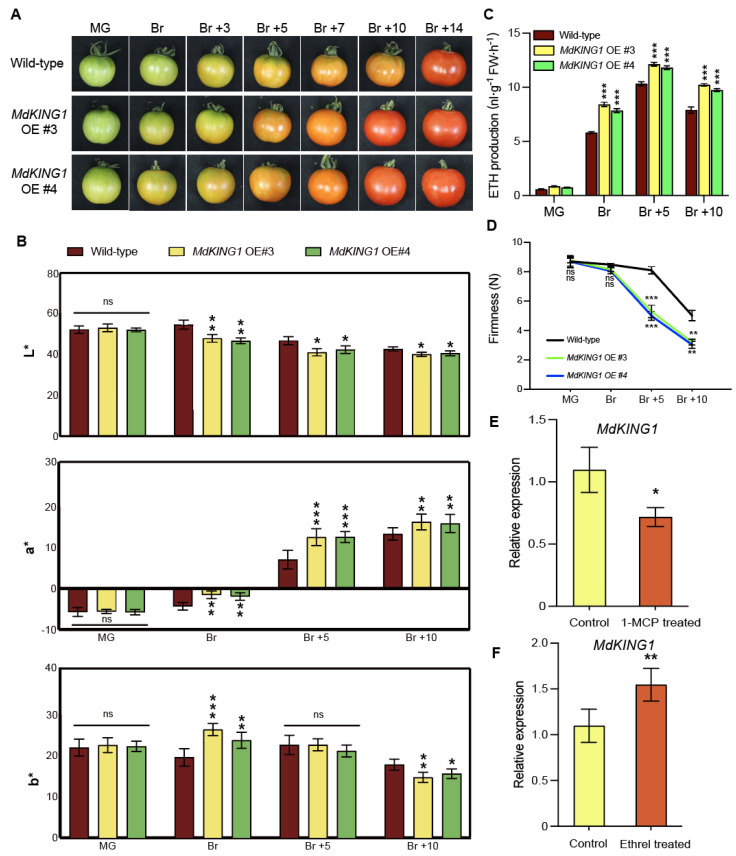
Overexpressing *MdKING1* in tomatoes accelerates fruit ripening. (**A**) Morphology of wild type (AC) and *MdKING1* OE tomato fruits at the mature green (MG) and breaker (Br) stages. (**B**) Color development in wild-type and overexpressing *MdKING1* tomato fruits. Pericarp color (L*, a*, b*), measured with a handheld colorimeter using the CIE L*a*b* color system, L* indicates lightness, a* indicates red to green, and b* indicates yellow to blue. (**C**,**D**) Determination of ethylene (ETH) production and fruit firmness of wild-type and *MdKING1* OE tomato fruits at MG and Br stages. (**E**,**F**) Accumulation of the *MdKING1* gene transcripts in WT fruit after treatment with 1-MCP or ethrel. Error bars indicate SD (*n* = 15 in (**B**), *n* = 3 in (**C**–**F**)). Asterisks indicate the significant differences between the *MdKING1* OE fruits and the wild-type in each group (* *p* ≤ 0.05, ** *p* ≤ 0.01, *** *p* ≤ 0.001; ns, no significance).

To investigate the function of MdKING1 in fruit ripening, we introduced the full-length *MdKING1* coding sequence (CDS) into the background of the cultivar Alisa Craig (AC). We generated four stable *MdKING1* overexpression (OE) transgenic tomato plants (Supplemental Figure S1A,B). *MdKING1* OE #3/#4 transgenic tomato plants were used for the following phenotypic analysis. Since the reddish color of the fruit was a characteristic of tomato fruit ripening [33], we observed and recorded the ripening characteristics of wild-type (WT) and *MdKING1* OE lines at seven different ripening stages (MG, Br, Br +3 to 14). Figure 3A showed that *MdKING1* OE fruits accumulated pigments more rapidly than WT. At the Br stage, we clearly observed that the *MdKING1* OE transgenic fruits were the first to show a color breaker compared with WT. In Br +10 stage, the skin of *MdKING1* OE fruits turned red completely, compared with WT fruit (Br +14 stage), suggesting that *MdKING1* OE fruits could enter the fruit ripening stage earlier. Characteristic of fruit ripening is the degradation of chlorophyll and the synthesis and accumulation of the carotenoids, lycopene, and β-carotene. A colorimeter was used to measure the color of tomato fruits pericarp by the CIE L*a*b* color system [34]. The values L* (Lightness), a* (color index), and b* (yellowness index) are indicated by the ratio of white to black color, red to green, and yellow to blue, determined by the degradation of chlorophyll and the accumulation of carotenoids, such as β-carotene and lycopene, which produce the characteristic yellow, orange, and red coloration (Figure 3B). The a* and b* values in *MdKING1* OE transgenic tomato fruits from coloring to ripening stage, were significantly higher than those in WT, except the b* value in the Br +5 period, and the L* value was significantly lower than WT (Figure 3B).

The respiration rate and ethylene content of climacteric fruit increased significantly during ripening. Ethylene plays a vital role in fruit ripening [35], and the ripening process is accompanied by softening of the fruit [33]. To better observe whether the overexpression of *MdKING1* in tomatoes could promote fruit ripening, we determined the ethylene production and fruit firmness at four different developmental stages (MG, Br, Br +5, +10) in WT and *MdKING1* OE fruits. The results showed that there was no significant difference in ethylene content between *MdKING1* OE fruits and WT at the MG stage, but the ethylene content of *MdKING1* OE fruits was significantly higher than WT from Br to Br +10 stage (Figure 3C). During the development period from MG to Br +10, the firmness of *MdKING1* OE fruits was lower, which decreased significantly at Br +5 and +10 stages than WT (Figure 3D). 

To determine whether *MdKING1* was involved in the ethylene signaling pathway, exogenous ethylene and the ethylene perception inhibitor 1-methylcyclopropene (1-MCP) were used to treat WT tomato fruits at MG and Br stages of fruit ripening, respectively [36]. The results showed that the transcription level of the *MdKING1* gene in apple fruit was induced by ethylene but inhibited by 1-MCP (Figure 3E,F). Based on the above results, it could be hypothesized that the *MdKING1* gene was induced by exogenous ethylene treatment, and it may promote the early ripening of tomatoes by involving in the ethylene signaling pathway.

### 2.3. MdKING1 Affects Fruit Ripening through the Ethylene Pathway

**Figure 4:**
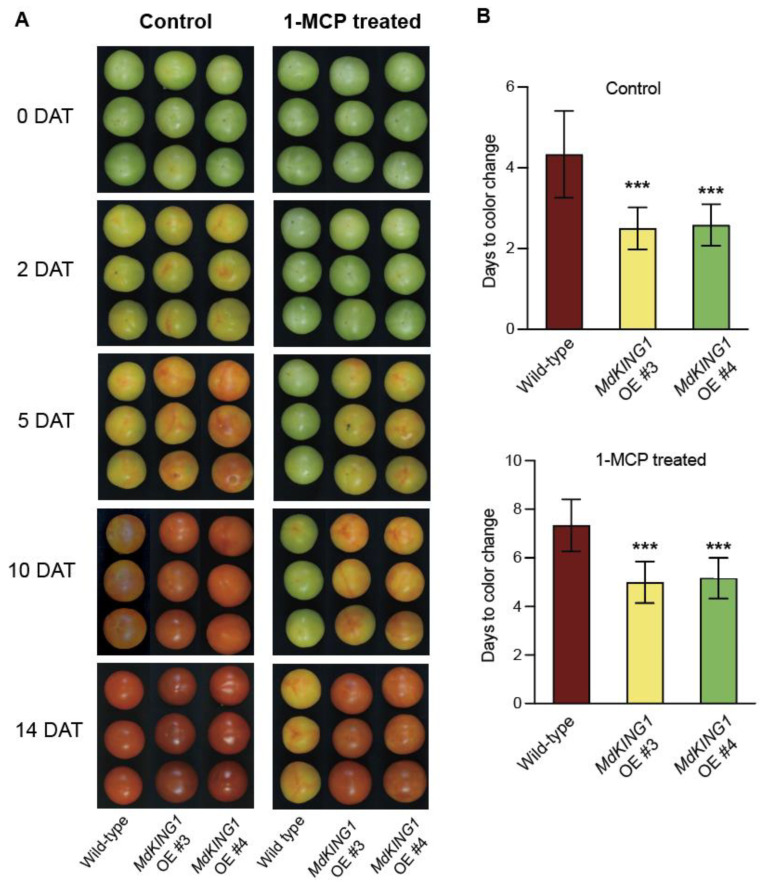
Overexpressing *MdKING1* in tomatoes reduced the fruits sensitivity to 1-MCP treatment. (**A**) Morphology of wild-type and *MdKING1* OE tomato fruits at 0, 2, 5, 10, and 14 days after control and 1-MCP treatment, respectively. DAT represents days after treatment. (**B**) Days from treatment beginning to color change stage in wild-type and *MdKING1* OE fruits under control conditions and 1-MCP treatment conditions, respectively. Air treatment was used as a control. Error bars indicate SD (*n* = 10). Asterisks indicate the significant differences between the *MdKING1* OE fruits and the wild-type in each group (*** *p* ≤ 0.001).

To further verify that the *MdKING1* gene affects tomato fruit ripening by responding to the ethylene signaling pathway, we performed 1-MCP treatment. The tomato fruits of WT and *MdKING1* OE at the Br stage were sampled and treated with 1.0 μL 1-MCP for 12 h, with air as the control group, then the phenotypes of fruits in the control were continuously observed and measured and treated at room temperature. After being treated with 1-MCP, the tomato fruits of *MdKING1* OE reached the color change 2–3 days earlier than the WT (Figure 4).

**Figure 5:**
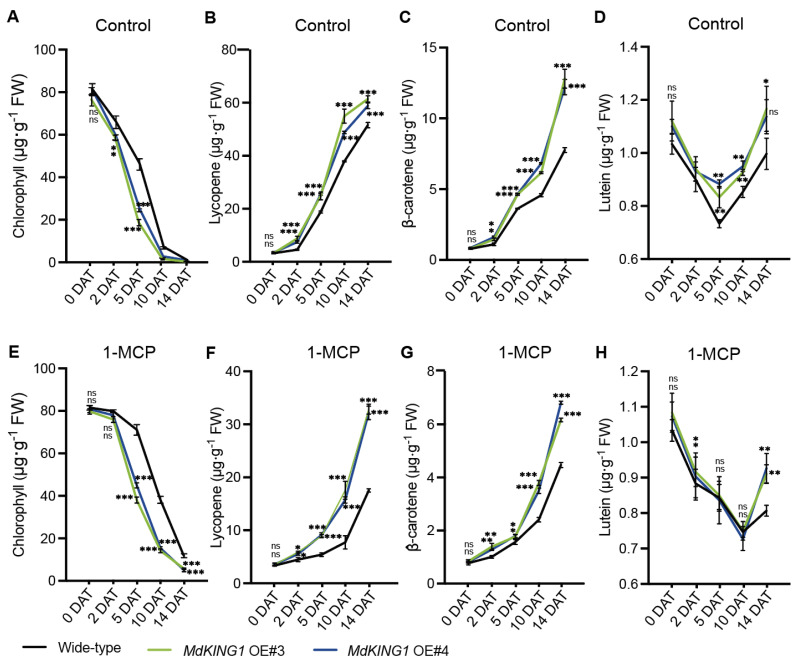
Several important indexes of fruit ripening in wild-type and *MdKING1* OE fruits. (**A**–**D**) Chlorophyll, lycopene, β-carotenoids, and lutein content at different days after treatment under control conditions, respectively. (**E**–**H**) Chlorophyll, lycopene, β-carotenoids, and lutein content at different days after 1-MCP treatment, respectively. Error bars indicate SD (*n* = 3). Asterisks indicate the significant differences between the *MdKING1* OE fruits and the wild-type in each group (* *p* ≤ 0.05, ** *p* ≤ 0.01, *** *p* ≤ 0.001; ns, no significance).

With the ripening of tomato fruits, carotenoids (including lycopene, β-carotenoids, and lutein) are gradually increased, and the chlorophyll is degraded [37]. To further investigate how *MdKING1* affects fruit ripening, we measured the content of chlorophyll, lycopene, β-carotenoids, and lutein at different stages. The results showed that the chlorophyll content of WT and *MdKING1* OE fruits decreased significantly under control conditions, and the decline rate of *MdKING1* OE fruits was faster than the WT (Figure 5A). Opposite to the change in chlorophyll in fruit development, the contents of lycopene, β-carotene, and lutein in both WT and *MdKING1* OE fruits gradually increased, and the content of carotenoids in *MdKING1* OE fruits increased rapidly compared to WT (Figure 5B–D).

Moreover, we measured the contents of chlorophyll and carotenoids in *MdKING1* OE and WT fruits at different time periods after 1-MCP treatment. We found the fruit ripening of both WT and *MdKING1* OE fruits was delayed, and the decline rate of chlorophyll content in WT fruit was slower than that of *MdKING1* OE (Figure 5E). Lycopene, *β*-carotene, and lutein gradually accumulated in WT and *MdKING1* OE with the prolongation of treatment time, and the increase rate of *MdKING1* OE fruit was faster than that of WT (Figure 5F–H).

Taken together, these results showed that the ethylene pathway blocked and could delay the fruit ripening of *MdKING1* OE lines, but ripened faster than WT, suggesting MdKING1 might be induced by ethylene and respond to the ethylene signaling pathway.

### 2.4. Transcriptomic Analysis of MdKING1 OE Fruits

**Figure 6:**
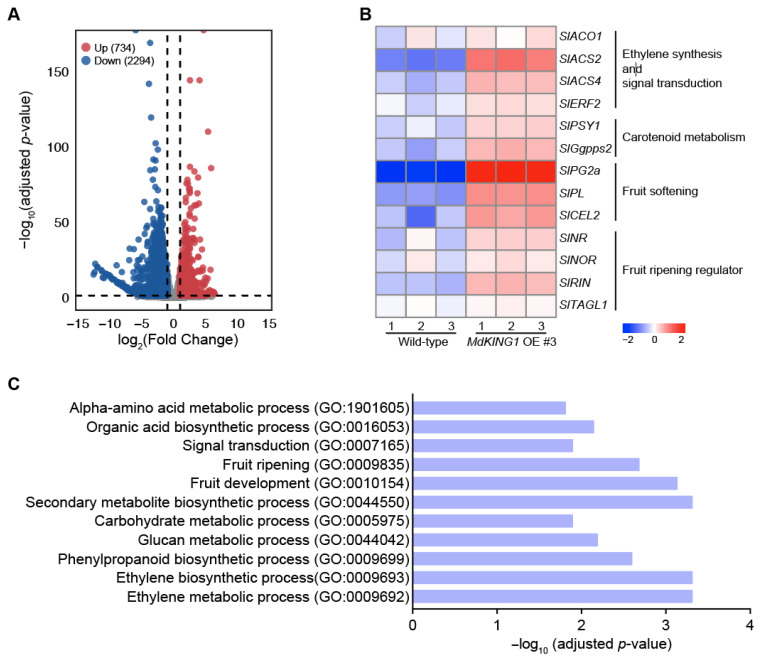
Transcriptome profiling analysis of wild-type and *MdKING1* OE fruits at Br stage. (**A**) Volcano map of differentially expressed genes (DEGs, log2 (fold change) ≥ 1) between wild-type and *MdKING1* OE fruits. Up and down refer to the upregulated and downregulated DEGs, respectively. (**B**) Heatmap analysis of the fruit ripening-related DEGs between wild-type and *MdKING1* OE fruits. (**C**) GO enrichment analysis of DEGs between wild-type and *MdKING1* OE fruits.

To further explore the mechanism of *MdKING1* in the fruit ripening process, we performed transcriptome deep sequencing (RNA-seq) analysis of WT and *MdKING1* OE fruits at the Br stage. Principal component analysis (PCA) shows a good correlation between the biological replicates (Supplemental Figure S2). As shown in Figure 6A, a total of 3028 differentially expressed genes (DEGs) were identified, of which 734 were upregulated and 2294 were downregulated in *MdKING1* OE fruits as compared with WT fruits. Consistent with the accelerated ripening in *MdKING1* OE lines, Gene Ontology (GO) enrichment analysis revealed that 3028 DEGs were significantly enriched in terms of association with the ethylene biosynthetic process (GO: 0009693), fruit development (GO: 0010154), fruit ripening (GO: 0009835), and the ethylene metabolic process (GO: 0009692) (Figure 6C). Furthermore, multiple metabolic pathways, including carbohydrate metabolic (GO: 0005975), amino acid metabolic (GO: 1901605), secondary metabolite (GO: 0044550), and signal transduction (GO: 0007165), were enriched (Figure 6C). The KEGG pathway analysis revealed the top 16 most enriched metabolic pathways, including flavonoid biosynthesis, phenylalanine metabolism, and galactose metabolism (Supplemental Figure S3).

**Figure 7:**
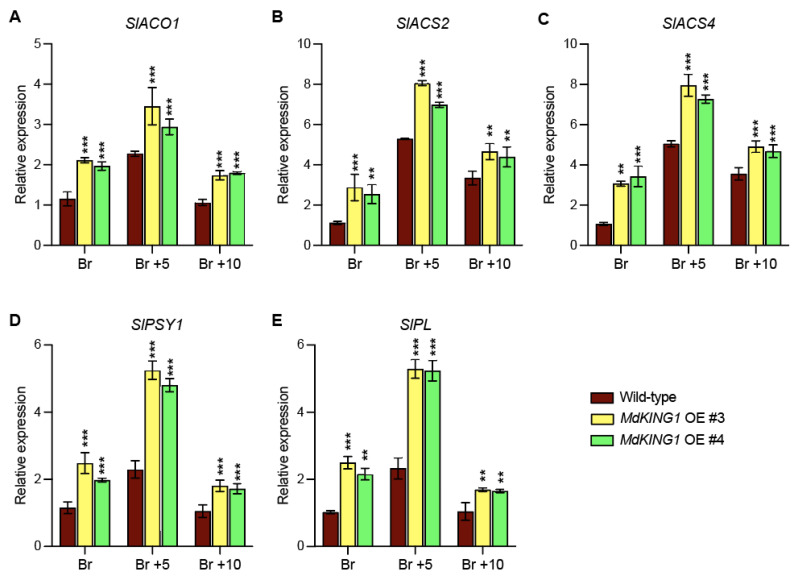
Expression profiles of ethylene biosynthesis, carotenoid biosynthesis, and fruit firmness-related genes affected by *MdKING1* overexpression in tomato fruits at Br, Br +5, and Br +10 stages. (**A**–**C**) The relative expression level of ethylene biosynthesis-related genes. (**D**) The relative expression level of carotenoid biosynthesis gene. (**E**) The relative expression level of cell wall metabolism-related gene. Br, Br +5, and Br +10 represent breaker stage, 5 days after breaker stage, and 10 days after breaker stage, respectively. Error bars indicate SD (*n* = 3). Asterisks indicate the significant differences between the *MdKING1* OE fruits and the wild-type in each group (** *p* ≤ 0.01, *** *p* ≤ 0.001).

We also investigated whether the ripening-related genes were modulated in *MdKING1* OE lines compared to WT. The heatmap of Figure 6B showed genes involved in ethylene synthesis and signal transduction (*SlACO1*, *SlACS2*, *SlACS4,* and *SlERF2*) [11,38], carotenoid metabolism (*SlPSY1* and *SlGgpps2*) [39,40], fruit softening (*SlPG2a*, *SlPL*, and *SlCEL2*) [41,42], and the key fruit ripening regulator (*SlNR*, *SlNOR*, *SlRIN*, and *SlTAGL1*) [43,44]; the expression level of all these genes was significantly increased in the *MdKING1* OE lines compared to that of WT. In addition, we verified the expression levels of *SlPL*, *SlPSY1*, *SlACO1*, *SlACS2*, and *SlACS4*, which were the DEGs in WT and *MdKING1* OE lines at the Br stage (Figure 7), the transcript levels of these five genes were significantly higher in *MdKING1* OE lines than WT, consistent with RNA-seq data. Finally, we also detected the expression levels of these five genes at other development stages to determine the role of *MdKING1* in promoting fruit ripening. As expected, the expression of all these genes in *MdKING1* OE lines was also higher than WT at Br +5 and +10 stages, and these genes were more highly expressed at the Br +5 stage than the other stages (Figure 7). In summary, these results suggest that *MdKING1* could affect the expression of genes related to fruit firmness as well as carotenoid and ethylene synthesis, thereby affecting fruit ripening.

## 3. Materials and Methods

### 3.1. Plant Materials and Growth Conditions

The tomato (*Solanum lycopersicum* cv. Alisa Craig, AC) was used as the transgenic materials and wild-type (WT). WT and transgenic lines were cultivated in standard greenhouses to ensure consistency of growth conditions. (Yangling City, Shaanxi Province, China) with natural light.

### 3.2. Generation of Transgenic Plants

The full-length *MdKING1* coding sequence (CDS) was amplified from apple (*Malus* × *domestica* cv. Golden Delicious) cDNA using the specific primer (Supplemental Table S1) into pENTR then introduced into the plant overexpression vector pK7WGF2. The resulting *pro35S*, *MdKING1*, was transformed into *Agrobacterium tumefaciens* strain GV3101, then transformed into tomato cultivar AC by Agrobacterium-mediated transformation. During tomato infection, tomato seeds were first surface sterilized with 75% alcohol and sodium hypochlorite solution, followed by immersion cleaning in sterilized distilled water to obtain sterile materials. After that, the sterilized seeds were spread on 1/2 MS solid agar medium (pH = 5.8) and left to germinate, and the cotyledons were assessed for Agrobacterium infection. Kanamycin was used for selecting stable transformants based on their resistance. Four independent T_2_ homozygous progeny were used to identify transgenic materials, in which *MdKING1* OE #3 and *MdKING1* OE #4 were used for phenotypic characterization.

### 3.3. Color Measurement

A WR-10QC colorimeter (Shenzhen Weifu Photoelectric Technology Co., Ltd., Shenzhen, China) with the CIE L*a*b* color system was chosen for the pericarp color assay [34]. At least fifteen biological replicates were used for each assay.

### 3.4. Ethrel and 1-Methylcyclopropene Treatment

Referring to previous studies [39], apple and tomato fruits were harvested at green stage and treated with 0.4% ethrel for 10 min, double-distilled water (DDW) as a control, then dried and transferred to room temperature conditions. The WT and *MdKING1* OE tomato fruits at breaker (Br) stage and color-turning apples were treated with 1.0 μL L^−1^ ethylene signaling inhibitor 1-methylcyclopropene (1-MCP) for 12 h in a sealed container. After treatment, peel tissue was sliced and frozen in liquid nitrogen for RNA extraction and qRT-PCR verification. For each treatment, there were three biological replicates from the independent sample.

### 3.5. Determination of Ethylene Content

WT and transgenic tomato fruits were harvested at MG, Br, Br +5, and Br +10 stages, then placed in the open container for 2 h. Then, these fruits were placed in a sealed container at room temperature for 24 h. Measurement of collected sealed container gases (1 mL headspace gas) was carried out using an Agilent gas chromatograph (GC). Samples were compared with reagent-grade ethylene standards of known concentration and normalized for fruit weight. The experiment was performed with three biological replications.

### 3.6. Determination of Carotenoid Content

Carotenoids of tomato fruits at MG, Br, Br +5, and +10 stages from *MdKING1* OE and WT were extracted as previously described [45], and 3 independent extractions were performed. The whole process of carotene extraction was carried out at low temperature, and light was avoided. Carotenoids were identified, and the relative contents were determined using high-performance liquid chromatography (HPLC) (LC-2030CD, Shimadzu, Kyoto, Japan) as previously described [45].

### 3.7. Measurement of Fruit Firmness

Ten fruits of each WT and transgenic tomato fruit were harvested at the MG, Br, Br +5, and +10 stages; their firmness was measured at five directions along the equatorial surface of the fruits with a fruit-firmness tester (GY-3, TOP Instrument, Zhejiang, China); and their average value was calculated. The experiment was performed with ten biological replications.

### 3.8. RNA Extraction and qRT-PCR Analysis

Total RNA was extracted from tomato fruits using the CTAB method [46,47]. After treatment with RNase-free DNase I (Thermo Fisher Scientific, Waltham, MA, USA), RNA was quantified using a NanoDrop One spectrophotometer (Thermo Fisher Scientific, Waltham, MA, USA). The RevertAid First Strand cDNA Synthesis Kit (Thermo Fisher Scientific, Waltham, MA, USA) was used to reverse transcribed 1 microgram of total RNA into first-strand cDNA. Detection of gene transcript levels was performed with ChamQ Universal SYBR qPCR Master Mix (C601, Vazyme Biotech Co., Ltd, Nanjing, China) on the CFX96 real-time system (Bio-Rad, Hercules, CA, USA). The relative expression was calculated using the 2^−ΔΔCt^ method with tomato *Actin* gene (*Solyc03g078400*) as an internal reference gene. The primers used are listed in Supplemental Table S1.

### 3.9. Sequence and Phylogenetic Analysis

The full-length protein sequence of KING1 in apple was obtained from the National Center for Biotechnology Information (NCBI, https://www.ncbi.nlm.nih.gov/, accessed on 15 February 2023), AtKINγ and other SnRK1 subunits in Arabidopsis was obtained from the Arabidopsis Information Resource (TAIR, https://www.arabidopsis.org/index.jsp, accessed on 15 February 2023), and SlKING1 in tomato was obtained from the Plant Genomics Resource (JGI, https://phytozome-next.jgi.doe.gov/, accessed on 18 February 2023). Sequence alignments were performed using the MUSCLE program (https://www.ebi.ac.uk/Tools/msa/muscle/, accessed on 18 February 2023), and the domain was predicted using the SMART program (version 9.0) (http://smart.embl-heidelberg.de/, accessed on 18 February 2023). The phylogenetic tree was inferred from the neighbor-joining method with 2000 bootstrap replications and the Poisson correction method in the MEGA 11 program [47].

### 3.10. Transcriptome Analysis

For transcriptomic analysis, the WT and *MdKING1* OE #3 fruits at the Br stage were selected as the experimental materials, and each sample had three biological replicates. The total RNA extraction and cDNA synthesis were performed as previously described [46,48]. Libraries were produced using the NEBNext^®^ UltraTM RNA Library Prep Kit (New England Biolabs, MA, USA), with which they were sequenced by using the Illumina HiSeq platform at Novogene (Beijing, China). Clean reads were aligned to the tomato reference genome (SL4.0, https://solgenomics.net/, accessed on 14 March 2023) with HISAT2 v2.1.0 (http://daehwankimlab.github.io/hisat2/, accessed on 14 March 2023). SAMTools v1.9 was utilized for subsequent data processing including BAM conversion, sorting, and indexing. Gene reads were detected by htseq-count in HTSeq v0.13.5 using the ITAG4.0 gene annotation file. Differentially expressed genes (DEGs) were identified based on a threshold of adjusted *p*-value < 0.05 and an absolute value of log2 (fold change) > 1 with the R package DESeq2 v1.20.1. Gene Ontology (GO) enrichment analysis was performed for the accession numbers with DEGs via g:Profiler [49]. KEGG enrichment analysis was performed using the KOBAS (version 3.0) software (http://bioinfo.org/kobas/genelist/, accessed on 20 March 2023).

### 3.11. Statistical Analysis

Significance analysis of corresponding experimental data was conducted using GraphPad Prism 8.0 software. Student’s two-tailed *t* test (* *p* ≤ 0.05, ** *p* ≤ 0.01, and *** *p* ≤ 0.001) was used for pairwise comparisons statistical analysis.

## 4. Discussion

Fruit ripening is governed by a complex network, and numerous genes have been identified as playing an important role in this development stage [10,50,51]. SnRKs are a large family of protein kinases, functioning as a heterotrimeric complex, including an α catalytic subunit and two regulatory subunits (KINβ and KINγ), which play an important role in plant development and stress response [20,52,53]. Previous studies have shown that *MhSnRK1* promotes fruit ripening through increasing starch and soluble sugars [29]. Yu [28] found that *Prunus persica* SNF1-related kinase α subunit (PpSnRK1a) interacts with RIN to regulate fruit ripening. However, little is known about the function of the SnRK γ subunit in fruit ripening. Here, we describe the role of the SnRKγ subunit (MdKING1) in fruit ripening, which may be regulated by influencing the expression of ripening-related genes and promoting carotenoid and ethylene content in response to ethylene signaling pathways. Previous research found that the ABA and SnRK signaling pathways could interact in order to regulate plant growth and metabolism [23]. ABA regulates non-climacteric fruit ripening [8] and increases the transcript levels of *ACS* and *ACO* in peach and tomato, respectively [54]. Xu [55] found the level of ABA accumulation increased at early fruit development of apple and then decreased at the fruit harvest stage. Similarly, the transcription level of *MdKING1* significantly increased during apple fruit ripening (Figure 1A). Meanwhile, the expression level of ethylene biosynthesis genes *SlACS2/4* and ethylene release showed the same trend in *MdKING1* OE tomato fruits (Figure 3C, Figure 6B and Figure 7). However, whether ABA participated in the fruit ripening which is mediated by *MdKING1* is not clear.

Previous studies showed that, in the early stages of fruit development (7 and 24 DAP), the relevant genes regulating fruit development in early-ripening “Geneva Early” were expressed earlier than in late-ripening “Hanfu” [32]. We observed that *MdKING1*, a homologous gene of *AtKINγ*, which contained four tandem cystathionine β–synthase (CBS) motifs (Figure 2A), has a higher expression level in early-cultivar “Geneva Early” apple than late-cultivar “Hanfu” at 7 and 24 days after pollination (DAP), and its transcription level significantly increased during apple fruit ripening (Figure 1A). Similarly, the expression of *MdKING1* in the five apple varieties of the early-ripening population was higher than that of the late-ripening population (Figure 1B,C). This result implied that MdKING1 may be an important factor affecting the fruit ripening of apples.

Tomato is an ideal model plant for studying climacteric fruit ripening [10]. During the ripening process, the respiration rate of climacteric fruit increases rapidly, accompanied by an increase in ethylene and carotenoid content [35,56,57]. Guo [58] found that *MdARD4* regulates the ethylene and carotenoid signaling pathways, increases ethylene and carotenoid content, and accelerates fruit ripening. Knockout of *SlMED25*, which is the positive ripening regulator, decreases ethylene and carotenoid content of the tomato fruit, thereby delaying the ripening of tomato fruit [38]. We observed that overexpression of *MdKING1* in tomatoes accelerated fruit ripening and made tomato fruits reach the Br stage earlier (Figure 3A and Figure 4A), and ethylene and carotenoid content of *MdKING1* OE tomato fruits was significantly higher than that of WT with the development of fruits (Figure 3C and Figure 5B–D). This result suggested that *MdKING1* may play an important role in fruit ripening via increasing ethylene and carotenoid content.

1-MCP is an ethylene signaling inhibitor that acts by irreversibly binding to ethylene receptors and blocking their normal binding to ethylene [59]. Exogenous application of 1-MCP can significantly suppress ethylene production and delay fruit ripening [60]. Wang [61] screened 12 *SlWRKY* genes by ethylene and 1-MCP treatment, which could respond to ethylene in tomatoes. In tomato fruits, the expression level of *SlACS2* was downregulated under 1-MCP treatment conditions, and the silencing of *SlACS2* inhibited fruit ripening by inhibiting ethylene, suggesting that SlACS2 was a positive regulator of tomato fruit ripening [39,62]. Similarly, our results showed that WT and *MdKING1* OE tomato fruits significantly delayed ripening after 1-MCP treatment. As the treatment process continued, *MdKING1* OE tomato fruits showed earlier ripening than WT (Figure 4). Under the treatment conditions of 1-MCP and ethylene, the expression level of *MdKING1* was inhibited and induced, respectively (Figure 3E,F). This also implied that MdKING1 may promote fruit ripening by responding to ethylene signaling. Fruit softening is a key component in an irreversible process during fruit ripening [63]. *SlLOB1* regulates fruit softening and promotes fruit ripening by upregulating cell-wall-related genes [64]. This is consistent with our finding that expression levels of fruit firmness-regulating genes (*SlPG2a*, *SlPL*, and *SlCEL2*) were significantly increased in *MdKING1* OE tomato fruits (Figure 7B), and *MdKING1* OE tomato fruits exhibited lower fruit firmness and earlier fruit ripening than WT (Figure 3). Taken together, these results suggest that *MdKING1* plays a positive regulatory role in fruit ripening.

Through RNA-seq analysis of *MdKING1* OE and WT fruits at the Br stage, the DEGs were enriched in several maturation-related biological processes, including “fruit ripening”, “fruit development”, “ethylene biosynthetic process”, and “ethylene metabolic process” (Figure 6C), which further indicated that *MdKING1* was involved in fruit ripening regulation through the ethylene pathway. Phytohormone ethylene has long been considered the main trigger of climacteric fruit ripening [38]. Ethylene biosynthesis involves two key biosynthetic enzymes, ACO and ACS [11]. In tomato fruit, *SlACO1* and *SlACO3* are gradually induced during ripening, and silencing of *SlACS2* inhibits fruit ripening by inhibiting ethylene [62,65]. In our results, ethylene-synthesis-related genes (*SlACO1*, *SlACS2*, *SlACS4*) and ethylene signal transduction-related genes (*SlERF2*) were significantly induced in the fruits of *MdKING1* OE (Figure 6B), which is also consistent with the faster ethylene production and earlier ripening in the fruits of *MdKING1* OE. Moreover, transcription factors (TFs) play an important role in fruit ripening [66]. MADS-RIN (or RIN) is a MADS-box transcription factor, and inhibition or knockout of MADS-RIN inhibits ethylene production because MADS-RIN directly targets *ACS2*, *ACS4*, and *ETR3/NR* [67] and directly regulates the expression of *ACO1*. Ripening of tomato fruits is controlled by a NAC transcription factor, such as non-ripening (NOR). Gene promoter analysis showed that genes involved in ethylene biosynthesis (*SlACS2* and *SlACS4*), color formation (*SlGgpps2* and *SlSGR1*), and cell wall metabolism (*SlPG2a*, *SlPL*, *SlCEL2*, and *SlXP1*) are direct targets of NOR-like1 [39]. TAGL1 is a critical transcription factor for fruit development and maturation, regulating chloroplast development and carotenoid accumulation in tomato fruits [68]. Similarly, in this study, we found that *SlNR*, *SlNOR*, *SlRIN*, and *SlTAGL1* expression in *MdKING1* OE fruit was significantly higher than that of WT (Figure 6B). However, *SlPL*, *SlPSY1*, *SlACO1*, *SlACS2*, and *SlACS4* had higher expression levels in the Br +5 stage than in Br and Br +10 stages (Figure 7), which may be determined by the temporal and spatial expression patterns of genes. Ultimately, the expression changes in this series of key ripening-related genes affect the fruit ripening process.

In summary, our results demonstrated that *MdKING1* played an important role in promoting fruit ripening by increasing fruit ethylene release and carotenoid accumulation. In addition, RNAseq analysis showed that *MdKING1* may promote ethylene-synthesis-related genes (*SlACO1*, *SlACS2*, and *SlACS4*), ethylene signal transduction-related genes (*SlERF2*), carotenoid metabolism-related genes (*SlPSY1* and *SlGgpps2*), and key regulators of fruit ripening (*SlNR*, *SlNOR*, *SlRIN*, and *SlTAGL1*) and ultimately promote fruit ripening. This study reveals the positive regulatory function of *MdKING1* in fruit ripening and provides a theoretical basis for fruit quality improvement and new cultivar breeding.

## Data Availability

The raw sequence data of RNA-seq have been deposited in the Genome Sequence Archive (NGDC, https://ngdc.cncb.ac.cn/gsa/), accessed on 30 July 2023, under the accession number BioProject ID: PRJCA018698.

## Supplementary Materials

The following supporting information can be downloaded at: https://www.mdpi.com/article/10.3390/plants12152848/s1, Figure S1. Identification of transgenic tomatoes. (A) Identification of transgenic tomatoes at DNA level; (B) RT-PCR analysis of *MdKING1* in wild-type and *MdKING1* OE plants. Figure S2. Principal component analysis of transcriptome data. WT represents wild-type, and OE #3 represents *MdKING1* OE #3. Each sample had three biological replicates. Figure S3. KEGG enrichment analysis of DEGs between wild-type and *MdKING1* OE fruits. The top 16 enriched KEGG pathways are exhibited. Table S1. List of primers used in this study.

## References

[1] Adams-Phillips L., Barry C., Giovannoni J. (2004). Signal transduction systems regulating fruit ripening. Trends Plant Sci..

[2] Zhai Y., Fan Z., Cui Y., Gu X., Chen S., Ma H. (2022). APETALA2/ethylene responsive factor in fruit ripening: Roles, interactions and expression regulation. Front. Plant Sci..

[3] Carrari F., Fernie A.R. (2006). Metabolic regulation underlying tomato fruit development. J. Exp. Bot..

[4] Seymour G.B., Østergaard L., Chapman N.H., Knapp S., Martin C. (2013). Fruit Development and Ripening. Annu. Rev. Plant Biol..

[5] Ji Y., Wang A. (2021). Recent Advances in Phytohormone Regulation of Apple-Fruit Ripening. Plants.

[6] Giovannoni J. (2001). Molecular biology of fruit maturation and ripening. Annu. Rev. Plant Physiol. Plant Mol. Biol..

[7] Palma J.M., Corpas F.J., del Río L.A. (2011). Proteomics as an approach to the understanding of the molecular physiology of fruit development and ripening. J. Proteom..

[8] Li C., Jia H., Chai Y., Shen Y. (2011). Abscisic acid perception and signaling transduction in strawberry: A model for non-climacteric fruit ripening. Plant Signal. Behav..

[9] Zhou L., Tang R., Li X., Tian S., Li B., Qin G. (2021). N6-methyladenosine RNA modification regulates strawberry fruit ripening in an ABA-dependent manner. Genome Biol..

[10] Chen T., Qin G., Tian S. (2020). Regulatory network of fruit ripening: Current understanding and future challenges. New Phytol..

[11] Palma J.M., Freschi L., Rodríguez-Ruiz M., González-Gordo S., Corpas F.J. (2019). Nitric oxide in the physiology and quality of fleshy fruits. J. Exp. Bot..

[12] Wang A., Yamakake J., Kudo H., Wakasa Y., Hatsuyama Y., Igarashi M., Kasai A., Li T., Harada T. (2009). Null mutation of the *MdACS3* gene, coding for a ripening-specific 1-aminocyclopropane-1-carboxylate synthase, leads to long shelf life in apple fruit. Plant Physiol..

[13] Li T., Tan D., Liu Z., Jiang Z., Wei Y., Zhang L., Li X., Yuan H., Wang A. (2015). Apple *MdACS6* Regulates Ethylene Biosynthesis During Fruit Development Involving Ethylene-Responsive Factor. Plant Cell Physiol..

[14] Li T., Jiang Z., Zhang L., Tan D., Wei Y., Yuan H., Li T., Wang A. (2016). Apple (*Malus domestica*) MdERF2 negatively affects ethylene biosynthesis during fruit ripening by suppressing *MdACS1* transcription. Plant J..

[15] Oeller P.W., Lu M.-W., Taylor L.P., Pike D.A., Theologis A.A. (1991). Reversible Inhibition of Tomato Fruit Senescence by Antisense RNA. Science.

[16] Hoogstrate S.W., van Bussel L.J.A., Cristescu S.M., Cator E., Mariani C., Vriezen W.H., Rieu I. (2014). Tomato *ACS4* is necessary for timely start of and progression through the climacteric phase of fruit ripening. Front. Plant Sci..

[17] Chen Y.-T., Lee Y.-R., Yang C.-Y., Wang Y.-T., Yang S.-F., Shaw J.-F. (2003). A novel papaya ACC oxidase gene (*CP-ACO2*) associated with late stage fruit ripening and leaf senescence. Plant Sci..

[18] Guo H., Ecker J.R. (2004). The ethylene signaling pathway: New insights. Curr. Opin. Plant Biol..

[19] Broeckx T., Hulsmans S., Rolland F. (2016). The plant energy sensor: Evolutionary conservation and divergence of SnRK1 structure, regulation, and function. J. Exp. Bot..

[20] Baena-González E., Lunn J.E. (2020). SnRK1 and trehalose 6-phosphate—Two ancient pathways converge to regulate plant metabolism and growth. Curr. Opin. Plant Biol..

[21] Ramon M., Ruelens P., Li Y., Sheen J., Geuten K., Rolland F. (2013). The hybrid four-CBS-domain KINβγ subunit functions as the canonical γ subunit of the plant energy sensor SnRK1. Plant J..

[22] Punkkinen M., Denessiouk K., Fujii H. (2019). Arabidopsis KIN gamma subunit 1 has a potential to regulate activity of sucrose nonfermenting 1-related protein kinase 2s (SnRK2s) in vitro. Biol. Plant..

[23] Jossier M., Bouly J.P., Meimoun P., Arjmand A., Lessard P., Hawley S., Grahame Hardie D., Thomas M. (2009). SnRK1 (SNF1-related kinase 1) has a central role in sugar and ABA signalling in Arabidopsis thaliana. Plant J. Cell Mol. Biol..

[24] Cui F., Brosché M., Lehtonen M.T., Amiryousefi A., Xu E., Punkkinen M., Valkonen J.P., Fujii H., Overmyer K. (2016). Dissecting Abscisic Acid Signaling Pathways Involved in Cuticle Formation. Mol. Plant.

[25] Jia M., Li X., Wang W., Li T., Dai Z., Chen Y., Zhang K., Zhu H., Mao W., Feng Q. (2022). SnRK2 subfamily I protein kinases regulate ethylene biosynthesis by phosphorylating HB transcription factors to induce *ACO1* expression in apple. New Phytol..

[26] Zhou Y., Li Z., Zhu H., Jiang Y., Jiang G., Qu H. (2022). Energy homeostasis mediated by the LcSnRK1α-LcbZIP1/3 signaling pathway modulates litchi fruit senescence. Plant J. Cell Mol. Biol..

[27] Li G., Peng F., Zhang L., Shi X., Wang Z. (2010). Cloning and characterization of a SnRK1-encoding gene from Malus hupehensis Rehd. and heterologous expression in tomato. Mol. Biol. Rep..

[28] Yu W., Peng F., Xiao Y., Wang G., Luo J. (2018). Overexpression of *PpSnRK1α* in Tomato Promotes Fruit Ripening by Enhancing RIPENING INHIBITOR Regulation Pathway. Front. Plant Sci..

[29] Wang X., Peng F., Li M., Yang L., Li G. (2012). Expression of a heterologous SnRK1 in tomato increases carbon assimilation, nitrogen uptake and modifies fruit development. J. Plant Physiol..

[30] Meng D., Cao H., Yang Q., Zhang M., Borejsza-Wysocka E., Wang H., Dandekar A.M., Fei Z., Cheng L. (2023). SnRK1 kinase-mediated phosphorylation of transcription factor bZIP39 regulates sorbitol metabolism in apple. Plant Physiol..

[31] Velasco R., Zharkikh A., Affourtit J., Dhingra A., Cestaro A., Kalyanaraman A., Fontana P., Bhatnagar S.K., Troggio M., Pruss D. (2010). The genome of the domesticated apple (*Malus* × *domestica* Borkh.). Nat. Genet..

[32] Yue Q., He J., Yang X., Cheng P., Khan A., Shen W., Song Y., Wang S., Ma F., Guan Q. (2023). Transcriptomic Analysis Revealed the Discrepancy between Early-Ripening ‘Geneva Early’ and Late-Ripening ‘Hanfu’ Apple Cultivars during Fruit Development and Ripening. Horticulturae.

[33] Changwal C., Shukla T., Hussain Z., Singh N., Kar A., Singh V.P., Abdin M.Z., Arora A. (2021). Regulation of Postharvest Tomato Fruit Ripening by Endogenous Salicylic Acid. Front. Plant Sci..

[34] Li S., Zhu B., Pirrello J., Xu C., Zhang B., Bouzayen M., Chen K., Grierson D. (2020). Roles of RIN and ethylene in tomato fruit ripening and ripening-associated traits. New Phytol..

[35] Paul V., Pandey R. (2014). Role of internal atmosphere on fruit ripening and storability-a review. J. Food Sci. Technol..

[36] Sisler E.C., Serek M. (1997). Inhibitors of ethylene responses in plants at the receptor level: Recent developments. Physiol. Plant..

[37] Liu L.H., Zabaras D., Bennett L.E., Aguas P., Woonton B.W. (2009). Effects of UV-C, red light and sun light on the carotenoid content and physical qualities of tomatoes during post-harvest storage. Food Chem..

[38] Deng H., Chen Y., Liu Z., Liu Z., Shu P., Wang R., Hao Y., Su D., Pirrello J., Liu Y. (2022). SlERF.F12 modulates the transition to ripening in tomato fruit by recruiting the co-repressor TOPLESS and histone deacetylases to repress key ripening genes. Plant Cell.

[39] Gao Y., Wei W., Zhao X., Tan X., Fan Z., Zhang Y., Jing Y., Meng L., Zhu B., Zhu H. (2018). A NAC transcription factor, NOR-like1, is a new positive regulator of tomato fruit ripening. Hortic. Res..

[40] Ampomah-Dwamena C., Tomes S., Thrimawithana A.H., Elborough C., Bhargava N., Rebstock R., Sutherland P., Ireland H., Allan A.C., Espley R.V. (2022). Overexpression of *PSY1* increases fruit skin and flesh carotenoid content and reveals associated transcription factors in apple (Malus × domestica). Front. Plant Sci..

[41] Wang D., Samsulrizal N.H., Yan C., Allcock N.S., Craigon J., Blanco-Ulate B., Ortega-Salazar I., Marcus S.E., Bagheri H.M., Perez Fons L. (2019). Characterization of CRISPR Mutants Targeting Genes Modulating Pectin Degradation in Ripening Tomato. Plant Physiol. Biochem..

[42] Llop-Tous I., Domínguez-Puigjaner E., Palomer X., Vendrell M. (1999). Characterization of two divergent endo-beta-1,4-glucanase cDNA clones highly expressed in the nonclimacteric strawberry fruit. Plant Physiol..

[43] Vrebalov J., Ruezinsky D.M., Padmanabhan V., White R., Medrano D.R., Drake R., Schuch W.W., Giovannoni J.G. (2002). A MADS-Box Gene Necessary for Fruit Ripening at the Tomato Ripening-Inhibitor (Rin) Locus. Science.

[44] Itkin M., Seybold H., Breitel D., Rogachev I., Meir S., Aharoni A. (2009). TOMATO AGAMOUS-LIKE 1 is a component of the fruit ripening regulatory network. Plant J..

[45] Fantini E., Falcone G., Frusciante S., Giliberto L., Giuliano G. (2013). Dissection of Tomato Lycopene Biosynthesis through Virus-Induced Gene Silencing. Plant Physiol..

[46] Chang S., Puryear J., Cairney J. (1993). A simple and efficient method for isolating RNA from pine trees. Plant Mol. Biol. Report..

[47] Niu C., Jiang L., Cao F., Liu C., Guo J., Zhang Z., Yue Q., Hou N., Liu Z., Li X. (2022). Methylation of a MITE insertion in the *MdRFNR1-1* promoter is positively associated with its allelic expression in apple in response to drought stress. Plant Cell.

[48] Cheng P., Yue Q., Zhang Y., Zhao S., Khan A., Yang X., He J., Wang S., Shen W., Qian Q. (2023). Application of γ-aminobutyric acid (GABA) improves fruit quality and rootstock drought tolerance in apple. J. Plant Physiol..

[49] Raudvere U., Kolberg L., Kuzmin I., Arak T., Adler P., Peterson H., Vilo J. (2019). g:Profiler: A web server for functional enrichment analysis and conversions of gene lists (2019 update). Nucleic Acids Res..

[50] Asif M.H., Lakhwani D., Pathak S., Gupta P., Bag S.K., Nath P., Trivedi P.K. (2014). Transcriptome analysis of ripe and unripe fruit tissue of banana identifies major metabolic networks involved in fruit ripening process. BMC Plant Biol..

[51] Li S., Li K., Ju Z., Cao D., Fu D., Zhu H., Zhu B., Luo Y. (2016). Genome-wide analysis of tomato NF-Y factors and their role in fruit ripening. BMC Genom..

[52] Hedbacker K., Carlson M. (2008). SNF1/AMPK pathways in yeast. Front. Biosci..

[53] Halford N.G., Hey S., Jhurreea D., Laurie S., McKibbin R.S., Paul M., Zhang Y. (2003). Metabolic signalling and carbon partitioning: Role of Snf1-related (SnRK1) protein kinase. J. Exp. Bot..

[54] Li S., Chen K., Grierson D. (2021). Molecular and Hormonal Mechanisms Regulating Fleshy Fruit Ripening. Cells.

[55] Xu J., Yan J., Li W., Wang Q., Wang C., Guo J., Geng D., Guan Q., Ma F. (2020). Integrative Analyses of Widely Targeted Metabolic Profiling and Transcriptome Data Reveals Molecular Insight into Metabolomic Variations during Apple (*Malus domestica*) Fruit Development and Ripening. Int. J. Mol. Sci..

[56] Saladié M., Matas A.J., Isaacson T., Jenks M.A., Goodwin S.M., Niklas K.J., Xiaolin R., Labavitch J.M., Shackel K.A., Fernie A.R. (2007). A reevaluation of the key factors that influence tomato fruit softening and integrity. Plant Physiol..

[57] Khoo H.E., Prasad K.N., Kong K.W., Jiang Y., Ismail A. (2011). Carotenoids and their isomers: Color pigments in fruits and vegetables. Molecules.

[58] Guo T., Zhang X., Li Y., Liu C., Wang N., Jiang Q., Wu J., Ma F., Liu C. (2020). Overexpression of *MdARD4* Accelerates Fruit Ripening and Increases Cold Hardiness in Tomato. Int. J. Mol. Sci..

[59] Phuong N., Le T., Vissenaekens H., Gonzales G.B., Camp J., Smagghe G., Raes K. (2019). In vitro antioxidant activity and phenolic profiles of tropical fruit by-products. Int. J. Food Sci. Technol..

[60] Yang Y.Y., Shan W., Kuang J.F., Chen J.Y., Lu W.J. (2020). Four HD-ZIPs are involved in banana fruit ripening by activating the transcription of ethylene biosynthetic and cell wall-modifying genes. Plant Cell Rep..

[61] Wang L., Zhang X.L., Wang L., Tian Y., Jia N., Chen S., Shi N.B., Huang X., Zhou C., Yu Y. (2017). Regulation of ethylene-responsive SlWRKYs involved in color change during tomato fruit ripening. Sci. Rep..

[62] Yang Y., Zheng Y., Liu C., Chen L., Ma J., Sheng J., Shen L. (2016). Inhibition of nitric oxide synthesis delayed mature-green tomato fruits ripening induced by inhibition of ethylene. Sci. Hortic..

[63] Shi Y., Li B.-J., Grierson D., Chen K.-S. (2023). Insights into cell wall changes during fruit softening from transgenic and naturally occurring mutants. Plant Physiol..

[64] Shi Y., Vrebalov J., Zheng H., Xu Y., Yin X., Liu W., Liu Z., Sorensen I., Su G., Ma Q. (2021). A tomato LATERAL ORGAN BOUNDARIES transcription factor, *SlLOB1*, predominantly regulates cell wall and softening components of ripening. Proc. Natl. Acad. Sci. USA.

[65] Alexander L., Grierson D. (2002). Ethylene biosynthesis and action in tomato: A model for climacteric fruit ripening. J. Exp. Bot..

[66] Wu J., Fu L., Yi H. (2016). Genome-Wide Identification of the Transcription Factors Involved in Citrus Fruit Ripening from the Transcriptomes of a Late-Ripening Sweet Orange Mutant and Its Wild Type. PLoS ONE.

[67] Zhong S., Fei Z., Chen Y.R., Zheng Y., Huang M., Vrebalov J., McQuinn R., Gapper N., Liu B., Xiang J. (2013). Single-base resolution methylomes of tomato fruit development reveal epigenome modifications associated with ripening. Nat. Biotechnol..

[68] Liu G., Li C., Yu H., Tao P., Yuan L., Ye J., Chen W., Wang Y., Ge P., Zhang J. (2020). *GREEN STRIPE*, encoding methylated TOMATO AGAMOUS-LIKE 1, regulates chloroplast development and Chl synthesis in fruit. New Phytol..

