# Supplementary material for: Heterologous Overexpression of Apple MdKING1 Promotes Fruit Ripening in Tomato
Source: Plants (Basel). 2023 Aug 2;12(15):2848. doi: 10.3390/plants12152848 (PMC10421076; doi:10.3390/plants12152848)
Supplement: Supplementary file 1 [file plants-12-02848-s001.zip › plants-2530406-supplementary.pdf]

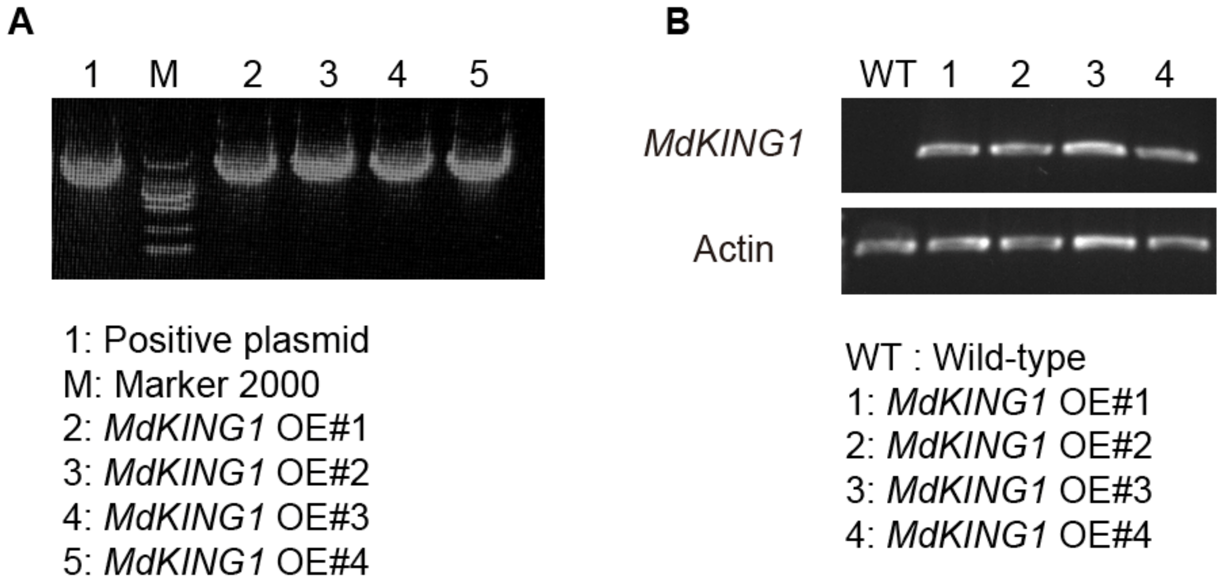

Supplemental Figure S1 Identification of transgenic tomatoes. (A) Identification of transgenic tomatoes at DNA level; (B) RT-PCR analysis of *MdKING1* in wild type and *MdKING1* OE plants.

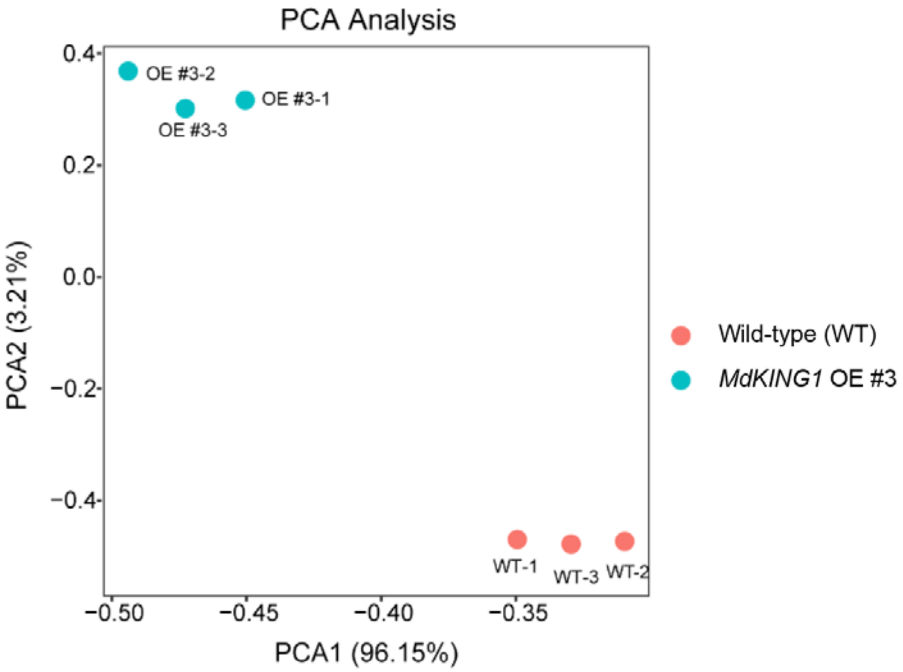

Supplemental Figure S2 Principal Component Analysis of Transcriptome Data. WT represents wild-type, and OE #3 represents *MdKING1* OE #3. Each sample has three biological replicates.

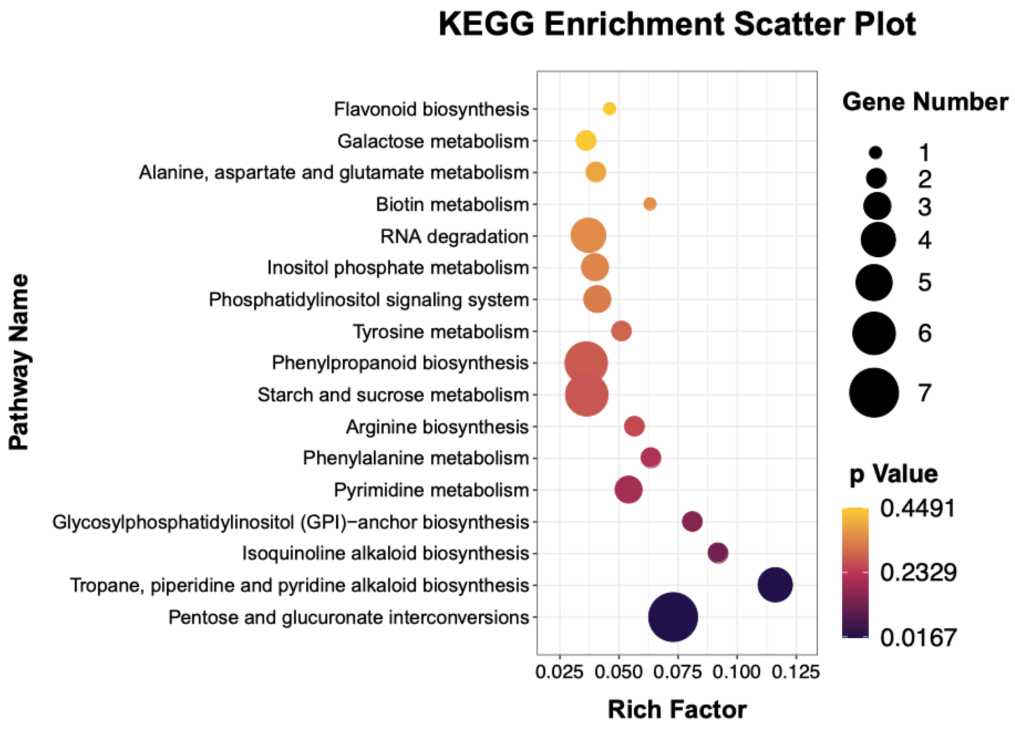

Supplemental Figure S3 KEGG enrichment analysis of DEGs between wild type and *MdKING1* OE fruits. The top 16 enriched KEGG pathways were exhibited.

Supplemental Table S1. Primers used in this study.

Name	Primer (5' to 3')	Purpose
SlActin-qF	CAGCAGATGTGGATCTCAAA	qRT-PCR
SlActin-qF	CTGTGGACAATGGAAGGAC	qRT-PCR
SlACS2-qF	AAACCCCAACGGAGTTATCC	qRT-PCR
SlACS2-qR	GCAATGGCCTTGAATGATTT	qRT-PCR
SlACS4-qF	TGGATTGCCTGAATTCACAA	qRT-PCR
SlACS4-qR	ATCACCAGGATCAGCCAAAC	qRT-PCR
SlPSY1-qF	GCTAATGACTCCCGAGAGAAGA	qRT-PCR
SlPSY1-qR	CGGGGTAATATATGATGCGTTT	qRT-PCR
SlPL-qF	ATTGGTGGAAGTGCTGATCC	qRT-PCR
SlPL-qR	GTGCATCCTCGTGCTTTGTA	qRT-PCR
SlACO1-qF	ATTCAGCCATCTCTTTGGTGAT	qRT-PCR
SlACO1-qR	ACTTGAACCCTGAACCTGTGTT	qRT-PCR
MdKING1-qF	ACAGATCCATCACGGCGAAG	qRT-PCR
MdKING1-qR	TCCCATCATCGTCCACAACG	qRT-PCR
MdKING1-CDS-qF	ATGGGTGTGCCGGCGGTGATGAC	Gene cloning
MdKING1-CDS-Qr	TCAAACCCTGCTGTTTTGGGGCAG CG	Gene cloning
MdKING1-pCAMBIA1305-qF	TACCCTCGAGACTAGTGTCGACAT GGGTGTGCCGGCGGTGATGAC	Vector construction
MdKING1-pCAMBIA1305-qR	TCCTGCAGGTGGATCCGAATTCA ACCCTGCTGTTTTGGGGCAGCG	Vector construction
